# Analysis of sperm separation protocols for isolating cryopreserved human spermatozoa

**DOI:** 10.1530/RAF-22-0133

**Published:** 2023-05-02

**Authors:** Alena J Hungerford, Hassan W Bakos, Robert J Aitken

**Affiliations:** 1 Priority Research Centre for Reproductive Science, University of Newcastle, Callaghan, NSW, Australia; 2 Monash IVF Group, Sydney, NSW, Australia

**Keywords:** cryopreservation, isolation, density gradient centrifugation, swim-up, electrophoresis, oxidative stress, DNA damage

## Abstract

**Graphical abstract:**

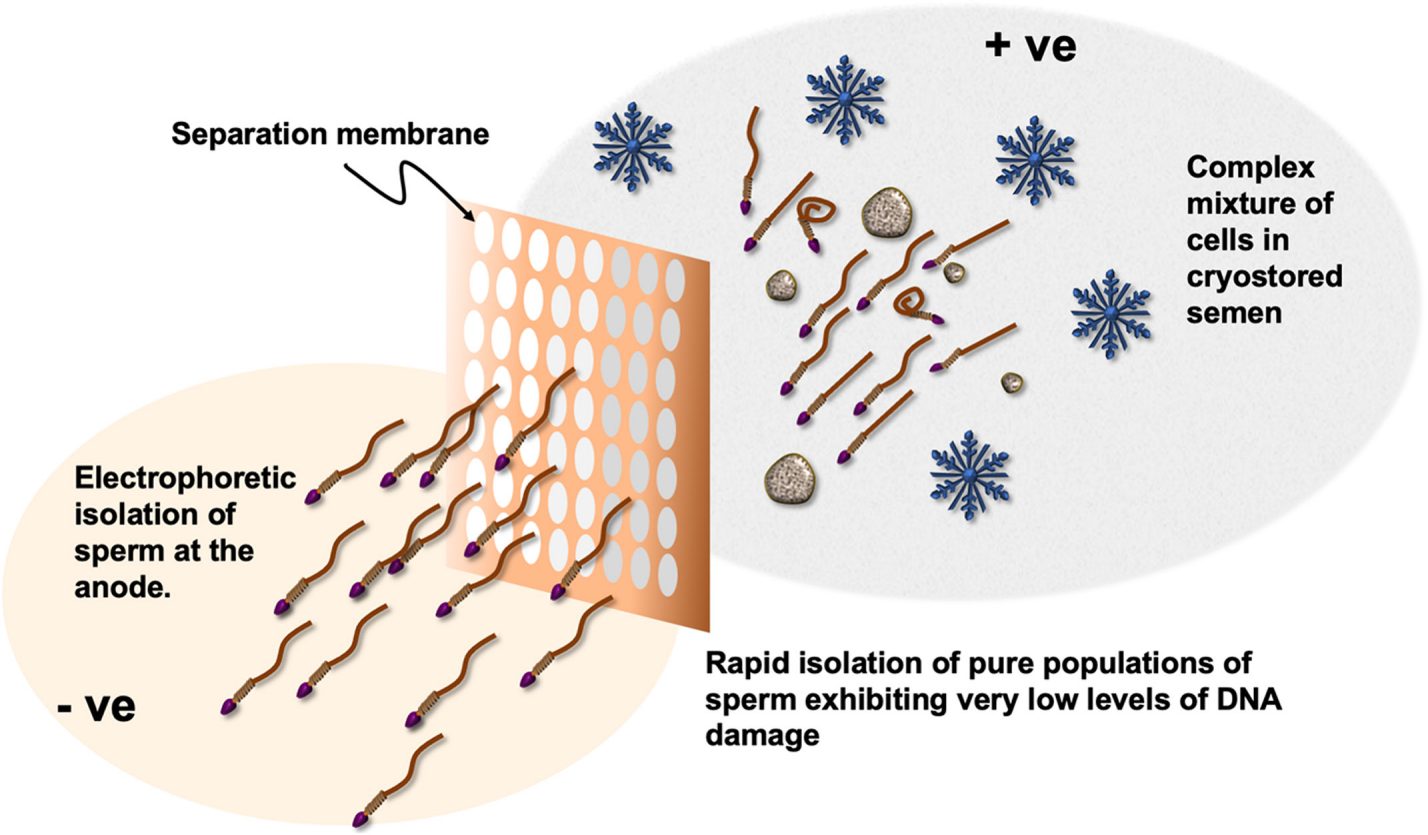

**Abstract:**

Sperm cryopreservation is a valuable tool for the long-term preservation of male fertility. Thus, determining the optimal technique for isolating spermatozoa post-thaw is vital to ensure recovery of the highest quality spermatozoa with minimal iatrogenic damage. This not only enhances the chances of successful conception but also reduces the risk of genetic damage in the embryo. To address this issue, human semen samples were cryopreserved using a slow freezing protocol and Quinn's Advantage™ Sperm Freeze medium. The samples were subsequently thawed and subjected to three types of sperm isolation procedures: direct swim-up, density gradient centrifugation, and electrophoretic separation using the Felix™ device. Cryopreservation led to the anticipated loss of sperm motility and vitality in association with increases in lipid peroxidation and DNA damage. Following sperm selection, all three isolation techniques resulted in an increase in sperm motility which was particularly evident with the swim-up and Felix™ procedures. The latter also significantly improved sperm vitality. There were no differences between sperm separation techniques with respect to morphology, and mitochondrial reactive oxygen species generation remained essentially unchanged when cell vitality was taken into account. By contrast, major differences were observed in DNA integrity and lipid aldehyde formation, where Felix™ isolated cells exhibiting significantly less DNA damage than the other isolation procedures as well as lower levels of 4-hydroxynonenal formation. Electrophoretic sperm isolation, therefore, offers significant advantages over alternative separation strategies, in terms of the quality of the gametes isolated and the time taken to achieve the isolation.

**Lay Summary:**

Long-term storage of sperm is vital to assisted reproductive technology because it permits the preservation of fertility that might be compromised as a result of factors such as chemotherapy or vasectomy. This goal can be achieved via cryopreservation – the freezing of cells to −196°C. When the sperm are subsequently required for conception, they must be carefully separated from the cryopreservation medium in a manner that maximizes the chances of successful conception and minimizes the risk of genetic defects in the offspring. In this paper, three isolation techniques were compared for their ability to separate ideal sperm from semen and media following cryopreservation. It was found that cryopreservation led to lower levels of motility and vitality and created higher levels of DNA and cell membrane damage. Of the three techniques compared, only cells separated on the basis of their size and electric charge (electrophoretic isolation) exhibited significantly lower levels of DNA fragmentation.

## Introduction

With 10–15% of couples encountering some form of infertility, investigations into the male and female factors contributing to impaired reproductive health have led to the creation of new and constantly improving assisted reproductive technology (ART) (Aitken 2020, Bosch *et al.* 2020). The male factor, in particular, makes a major contribution to the overall infertility burden carried by our species. It is a highly prevalent, multifaceted condition that may manifest itself in a wide variety of ways, including reduced sperm number, lowered sperm motility, abnormal sperm morphology, impaired capacitation and sperm-egg recognition, lowered vitality, DNA damage, and oxidative stress (Zini & Libman 2006, Aitken & Roman 2008, Lombardo *et al.* 2011, Aitken & Gibb 2022). It has become clear in recent years that oxidative stress is centrally involved in the aetiology of male infertility affecting both the functional competence of these cells and their genetic integrity (Jones *et al.* 1979, Aitken & Clarkson 1987, Alvarez *et al.* 1987, Kemal Duru *et al.* 2000, Aitken 2020, Barati *et al.* 2020, Aitken *et al.* 2022*a*,* b*). Reactive oxygen species (ROS) are largely created when the mitochondrial electron transport chain leaks electrons that, instead of achieving the controlled 4-electron reduction of ground state oxygen to water, partially reduce oxygen to create reactive oxygen intermediates such as superoxide anion and hydrogen peroxide. While ROS are necessary for low doses to drive critical biological processes such as sperm capacitation (Aitken *et al.* 2015), in excess, they primarily attack the fundamental building blocks of life (proteins, carbohydrates, lipids, and proteins) and disrupt the mitochondrial electron transport chain, creating a self-perpetuating cycle of ROS production that can eventually lead to cellular dysfunction and accelerated apoptosis (Storey 1997, Kemal Duru *et al.* 2000, Valko *et al.* 2007, Aitken *et al.* 2010, 2012, 2022*a*,*b*, Gharagozloo & Aitken 2011, Barati *et al.* 2020).

One area of ART where oxidative stress is known to have a major influence is sperm cryopreservation (Royere *et al.* 1996). This technology is being increasingly used as a tool to preserve male fertility, particularly in cancer patients where the deployment of powerful chemotherapeutic agents can have a devastating impact on fertility (Vutyavanich *et al.* 2010, Yumura *et al.* 2022). However, unfortunately, spermatozoa that have been through this process are extremely fragile, exhibiting low levels of motility and high levels of DNA damage in association with all the hallmarks of oxidative stress (Thomson *et al.* 2009, Raad *et al.* 2018, Ghantabpour *et al.* 2022). As a consequence, the methods used to isolate these cells following cryopreservation have to be considered with extreme care.

In this study, we have directly compared three sperm isolation procedures (semen swim-up, density gradient centrifugation (DGC), and electrophoresis using the Felix™ device) for their ability to isolate cryopreserved human spermatozoa with a minimum of damage (Aitken & Clarkson 1988, Ainsworth *et al.* 2005, Ainsworth & Aitken 2011, Vaughan & Sakkas 2019). The quality of the isolated cells was monitored by reference to sperm motility, vitality, morphology, mitochondrial ROS production, lipid aldehyde generation, and DNA fragmentation. The results emphasize the unique ability of the electrophoretic sperm isolation system to isolate cryostored human spermatozoa exhibiting both minimal DNA damage and relatively high levels of motility.

## Materials and methods

### Patients and donors

All experiments were conducted in accordance with protocols approved by the University of Newcastle Human Research and Ethics Committee as well as the Monash *in-vitro* fertilisation (IVF) Research Approval Committees and staff (H-2013-0319 and 200621). These approvals were issued in 2021. In line with the approved project proposal, all patients were informed that their samples would be used for research purposes and signed consent was given.

### Study design

The core of this study is based on 40 semen samples collected from either the University of Newcastle (*n* = 25) or Monash IVF (*n* = 15). The basic semen profiles for these samples are presented in Table 1. All of the subjects from the Newcastle and Sydney sites were of unknown fertility status at the time of analysis and about half (52% in Newcastle and 40% in Sydney) were normozoospermic according to WHO criteria (World Health Organization 2021). All other samples had at least one defective criterion in their profile. The age ranges for the subjects contributing samples to this analysis were 27–44 in Sydney and 18–75 in Newcastle. In addition to this core group, an additional 15 subjects were assessed by CASA to determine the impact of cryopreservation on the quality of sperm movement, while an additional 10 samples were analysed to examine the impact of cryostorage on the lipid peroxidation levels observed prior to sperm separation. Details of the semen profiles for these additional subjects are also given in Table 1. As indicated in Supplementary Table 1 (see section on supplementary materials given at the end of this article), the source of the material (Newcastle donors or Monash IVF patients) did not have a significant impact on the changes observed in this study as a result of cryopreservation although the initial motility was higher in the Monash IVF samples. Importantly, the experimental core of this study involving the thawing of cryopreserved semen samples and their subsequent isolation using a variety of sperm preparation techniques was all conducted at the Newcastle site by a single researcher (AH).

**Table 1:** Attributes of semen quality for the samples used subjected to cryostorage. Data are presented as mean ± s.e.m.

Cohort	*n*	Sperm count (10^6^/mL)	Motility (%)	Morphology (% normal )	Vitality (%)
Core experimental group	40	65.2 ± 7.9	54.5 ± 3.0	6.9 ± 0.5	80.5 ± 2.2
Group for CASA	15	61.3 ± 15.2	66.4 ± 2.3	6.7 ± 0.9	80.6 ± 2.5
Group for 4HNE	10	65.9 ± 8.6	62.6 ± 4.7	7.2 ± 0.8	75.9 ± 7.6

An initial semen profile (sperm motility, morphology, vitality, and count) was created from 1.5 mL aliquots of semen. The remaining aliquot was diluted with 1.5 mL of warmed cryoprotectant (Quinn’s Advantage^TM^ Sperm Freezing Medium, CooperSurgical, Ballerup, Denmark) and held for a maximum of 5 min, before being cryopreserved and stored at −196°C in a liquid nitrogen tank for a maximum of 4 months. Following cryopreservation, each sample was thawed for 15 min at room temperature, divided into four 0.75 mL aliquots, and isolated by semen swim-up, DGC, Felix™, or no isolation (control), ultimately generating suspensions of isolated spermatozoa in a conventional IVF medium, GIVF+ (Vitrolife, Gothenburg, Sweden). Following isolation, the spermatozoa were examined to determine their motility, morphology, mitochondrial ROS generation, lipid aldehyde formation, and levels of DNA damage.

### Semen profile

Sperm motility post-freeze was determined using a CASA system (Hamilton Thorne, IVOS II, Beverly, MA, USA) (World Health Organization 2021). Vitality was assessed at ×400 using the eosin method and counting 200 cells (World Health Organization 2021). Sperm morphology was measured by the careful examination of spermatozoa placed on pre-stained slides (Testsimplets Waldeck, GmbH, Münster, Germany) under oil immersion and scored at ×1000 magnification. A total of 200 cells were counted to determine the percentage of morphologically normal cells. Sperm counts were assessed using a Neubauer haemocytometer as described by the World Health Organisation (2021).

### Peroxidative damage

Peroxidation damage was monitored by analysing the spermatozoa for the lipid aldehyde, 4-hydroxynonenal (4-HNE) using a FACSCalibur flow cytometer (Becton Dickinson, Franklin Lakes, NJ, USA) with a 488-nm argon ion laser. Fluorescence measurements were made using 585/42-nm band-pass (red/FL-2), 530/30-nm band-pass (green/FL-1), and 670-nm long-pass (far red/FL-4) filters. Side-scatter and forward-scatter plots were used to gate relevant sperm cells and exclude debris. The data were acquired and analysed using CellQuest Pro software (Becton Dickinson) with at least 2000 events collected per sample. This process was commenced by staining the cells with a primary anti-4-HNE antibody (Molecular Probes) at a dilution of 49 µL Biggers, Whitten and Whittingham medium (BWW;Biggers *et al.* 1997) to 1 µL antibody. Once stained, the spermatozoa were incubated at 37°C for 30 min before centrifuging (500 ***g*** for 5 min) and resuspending in BWW. A secondary antibody (AlexaFluor-488 goat anti-rabbit IgG, Molecular Probes) was then added at a dilution of 99 µL BWW to 1 µL antibody and the mixture was incubated at 37°C for 10 min before centrifuging and resuspending in BWW.

### Mitochondrial reactive oxygen species

ROS generation by the sperm mitochondria was detected by flow cytometry using MitoSOX™ Red dye (MSR; Molecular Probes). This reagent was prepared at a dilution of 1 µL of 5 mM MSR in 249 µL BWW. Sytox® Green dye (SyG, Molecular Probes) was then added at a dilution of 1 µL 12.5 mM SyG in 249 µL BWW in order to differentiate live and dead cells (required because trace amounts of ethidium in the MSR probe can bind directly to the nuclei of dead cells with compromised plasma membrane integrity). As a positive control, arachidonic acid (AA; Sigma Aldrich) was added at a dilution of 1 µL 50 mM AA in 99 µL BWW because this fatty acid is known to stimulate mitochondrial ROS production by spermatozoa (Aitken *et al.* 2013); this positive control generated an average ± s.e.response in 91.3 ± 1.7% of cells. Once stained with MSR and SyG solutions, the spermatozoa were incubated at 37°C for 15 min before centrifugation and resuspension in BWW. The results were ultimately expressed as the percentage of live MSR-positive cells/all cells and percentage of live MSR-positive cells/live cells.

### DNA fragmentation

DNA integrity was studied using the Halo assay on spermatozoa that had been snap frozen and resuspended in BWW. This laboratory assay involved setting the spermatozoa in 0.65% agarose on slides which were then immersed in 0.08 M HCl for 7 min, followed by 100 mM DTT in Tris buffer 1 (4.84 g of Tris, 10 mL of 10% SDS, 10 mL of 0.5 M EDTA made up to 100 mL with MilliQ; pH 7.5) for 10 min. The slides were then immersed in Tris buffer 2 (4.84 g Tris, 11.69 g NaCl, 10 mL 10% SDS made up to 100 mL with MilliQ; pH 7.5) for 5 min, followed by immersion in Tris-Boric Acid-EDTA Buffer (TBE: 5.4 g Tris, 2.75 g boric acid, 2 mL of 0.5 M EDTA made up to 100 mL with MilliQ; pH 7.5) for 2 min. The slides were then passaged through increasing strengths of ethanol (70, 90, and 100% ethanol) allowing 2 min for each step. They were then air dried and stained with 4′,6-diamidine-2′-phenylindole dihydrochloride (DAPI, Sigma-Aldrich) solution for 10 min (1 µL of DAPI per 2 mL of phosphate-buffered saline (PBS: pH 7.4, 137 mM NaCl, 2.7 mM KCl, 8 mM Na_2_HPO_4_, and 2 mM KH_2_PO_4_) generating a final DAPI concentration of 1.8 µg/mL). The slides were finally rinsed with PBS, 30 µL of Mowiol ® (Sigma-Aldrich,) added and coverslips applied. For scoring purposes, the cells were classified into five categories: large halo, medium halo, small halo, no halo, or degraded sperm. The percentage of DNA-damaged spermatozoa was given by the percentage of cells falling into the small halo, no halo and degraded categories, as recommended (Gallegos *et al.* 2008).

### CASA assessment of sperm motility

The quality of sperm movement was assessed with a CASA system (Hamilton Thorne, IVOS II, Beverly, MA, USA) and the following criteria were assessed: total motility, progressive motility, curvilinear velocity (VCL; µm/s), average path velocity (VAP; µm/s), straight line velocity (VSL; µm/s), straightness (STR; VSL/VAP), linearity (LIN; VSL/VCL), and amplitude of lateral head displacement (ALH) using the following settings: minimum total count 200; kinematics: progressive STR (%) 80, progressive VAP (µm/s) 25, slow VAP (µm/s) 5, slow VSL (µm/s) 5, static VAP (µm/s) 0, and static VSL (µm/s) 0.

### Cryopreservation protocol

Cryostorage was achieved by the semen sample being slowly mixed in a 1:1 ratio with Quinn’s Advantage^TM^ Sperm Freezing Medium, a commercial HEPES-buffered salt solution containing human serum albumin, glycerol, sucrose, and gentamicin (Cooper Surgical), and then loaded into 0.5 mL straws (CBS High Security Sperm Straw with Tripartite Plug, IMV, L’Aigle, France: Conception Technology, CA) with the ends heat sealed. The straws were gradually cooled in liquid nitrogen by controlled freezing employed by the FreezeControl^TM^ system from Cryologic (Victoria, Australia). After freezing to −80°C, the straws were placed in goblets and stored at −196°C in a liquid nitrogen tank for a maximum of 4 months. Thawing was conducted by removing the straws from the tank and letting them acclimatise to room temperature in the laboratory over 15 min. After acclimatisation, the straw exterior was washed with distilled water, dried, cut, and poured into a Falcon tube. Once thawed, the sample was isolated within the next 15 min to avoid unnecessary damage from continued exposure to the cryoprotectant.

### Sperm isolation

Swim-up isolation from semen was conducted following protocols established at Monash IVF with minor modifications (Raad *et al.* 2021). For this procedure, an appropriate volume of semen (0.7 mL) was pipetted under 1 mL of GIVF+, which was then placed in a 37°C incubator for 30 min. The time was extended to a maximum 1 h for poor-quality samples. After 30 min, the top 500 µL of liquid was removed and pipetted into a separate tube.

DGC isolation was conducted using a two-step gradient according to the basic Monash IVF protocol with minor modifications (Popkiss *et al.* 2022). Semen (0.7 mL) was pipetted on top of the gradient and the contents were centrifuged at 300 ***g*** for 20 min, creating a pellet. The supernatant was removed and 2 mL of GIVF+ was added to the pellet to be centrifuged again at 300 ***g*** for 10 min. The final pellet was then resuspended in 500 µL GIVF+.

Electrophoretic isolation was conducted by utilising an electrophoretic device manufactured by Memphasys Ltd – the Felix™ system (https://www.memphasys.com/about-us/what-we-do-humans/). To use Felix™, a fresh cartridge was inserted into the console bay, then 4 mL of buffer media (G-Rinse, Vitrolife) pipetted into chambers 1 and 2, 1 mL of harvest media (GIVF+) pipetted into chamber 3, and finally 0.7 mL of undiluted semen pipetted into chamber 4. Once running, the entire separation process took 6 min. After separation, 0.5 mL of fractionated spermatozoa were removed from chamber 5.

No isolation was conducted as a control, whereby the semen mixture was centrifuged at 500 ***g***for 3 min, creating a pellet. The supernatant was removed and 1 mL of GIVF+ was added. The suspension was then centrifuged again at 500 ***g*** for 3 min and the final pellet was resuspended in 500 µL GIVF+. Such repeated washing steps were necessary as spermatozoa remaining in Quinn’s Advantage^TM^ following the thawing process would suffer rapid, irreparable damage.

### Statistical analysis

Statistical analysis of the data was performed using JMP® Pro 16.2.0. Initially, all numerical data were examined to determine the normality of their distribution using the Shapiro–Wilk test. If the data significantly deviated from a normal distribution, then both log_e_ and square root transformations were used in an attempt to correct the deviation from normality. The significance of differences between groups was examined using a one-way analysis of variance followed by an analysis of group means using the Tukey-Kramer HSD procedure. If transformation did not achieve a normal distribution, then groups were compared using the nonparametric Wilcoxon method. Results with a probability of *P* < 0.05 were considered significant.

## Results

### Semen cryopreservation

The quality of the original semen samples (*n* = 40) subjected to cryopreservation is presented in Table 1. Cryopreservation had the expected impact on motility, which was reduced from 54.5 ± 3.0% to 17.1 ± 2.4%; (*P* < 0.001) and vitality, which was decreased from 80.5 ± 2.2% to 37.1 ± 2.4 % (*P* < 0.001), while morphology remained essentially unchanged at 6.3 ± 0.5% normal. In addition, a small number of additional samples (*n* = 10) were examined to determine whether this decrease in motility and vitality following cryostorage was associated with a significant increase in 4-HNE formation, indicating the concomitant induction of lipid peroxidation (Table 1). This analysis demonstrated a significant increase in the percentage of 4-HNE positive cells as a consequence of cryopreservation from 22.0 ± 3.3% 4-HNE positive cells in the original semen to 47.0 ± 5.2% (*P* <0.01) immediately after thawing. Cryopreservation also increased the incidence of DNA damage from 21.4 ± 1.7% to 29.8 ± 3.2% (*P* <0.05); however, interestingly the generation of mitochondrial ROS remained statistically unchanged (28.3 ± 1.6% prior to freezing and 17.5 ± 1.6% following cryopreservation).

### Impact of sperm isolation technique following cryopreservation

For this analysis, 40 frozen-thawed samples were divided into 4 portions comprising an unselected control, and samples isolated by semen swim-up, DGC, and the Felix™ electrophoretic system. The attributes of the isolated spermatozoa were then assessed with reference to their recovery rate, motility, vitality, morphology, lipid peroxidation status, level of mitochondrial ROS generation, and DNA damage.

**Figure 1:**
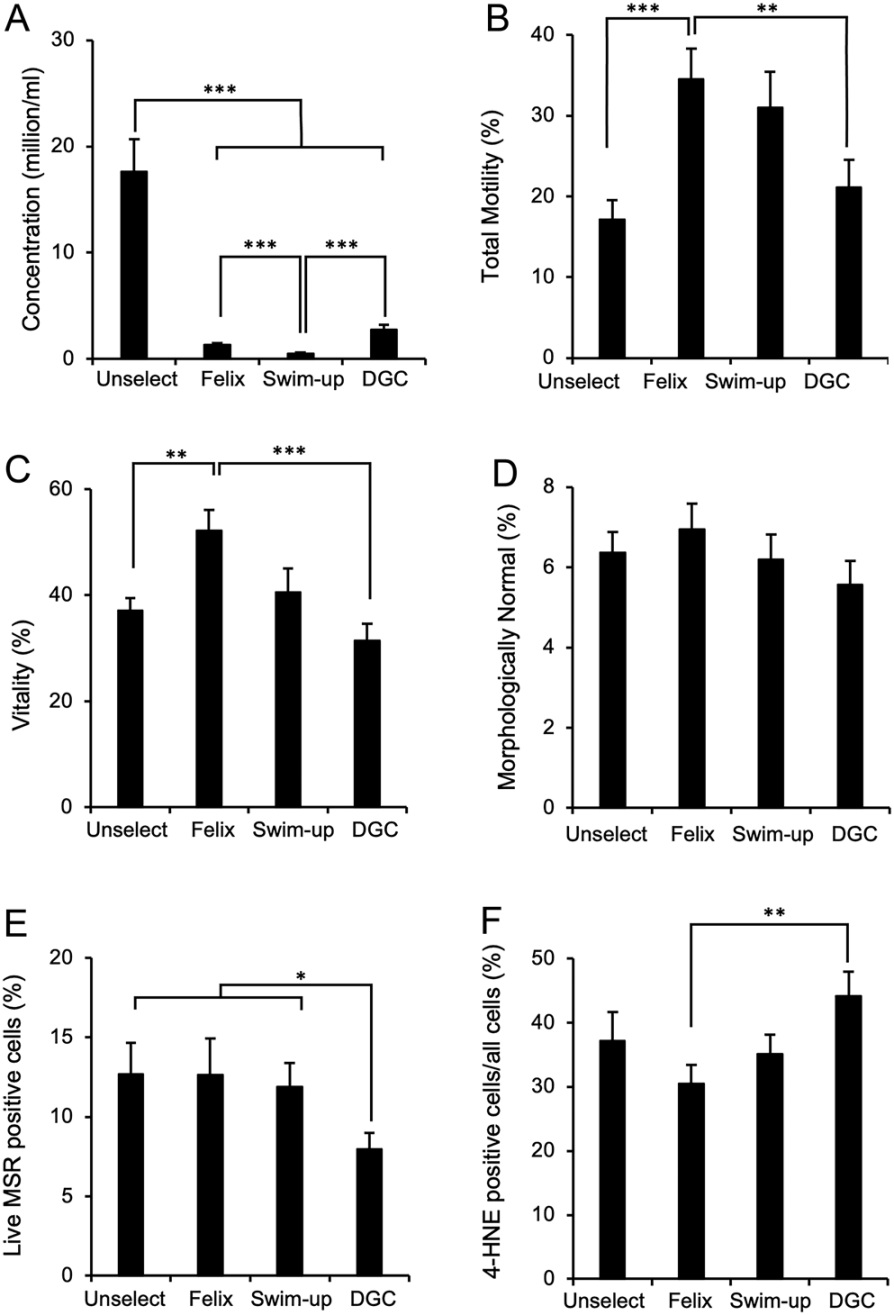
Impact of sperm isolation protocol on the quality of cryopreserved human spermatozoa. Following the freeze-thaw procedure, spermatozoa were recovered without any selection (Unselect) or following selection by the Felix™ electrophoretic system, swim-up, or DGC. (A) Concentration of recovered spermatozoa in millions/mL. (B) Percentage of motile cells. (C) Percentage of vital cells. (D) Percentage of cells with normal morphology. (E) Mitochondrial ROS generation measured with MitoSOX Red (MSR). (F) Lipid aldehyde formation expressed as the percentage of 4-HNE positive cells. Data presented as means ± s.e.; *n* = 40. *P < 0.05, **P < 0.01, ***P < 0.001.

Not surprisingly, all of the sperm selection techniques generated a reduced number of spermatozoa compared with the unselected control (*P* <0.001). With respect to the individual sperm isolation protocols, ‘semen swim-up’ generated the lowest concentration of spermatozoa, resulting in sperm concentrations that were significantly lower than the recovery rates observed with Felix™ (*P* <0.001), that were, in turn, significantly lower than those achieved DGC, which recovered the highest number of spermatozoa (*P* <0.001) (Fig. 1A). In the context of sperm motility (Fig. 1B), Felix™ was the only isolation procedure to significantly increase total motility above the unselected control level (*P* <0.05 by ANOVA and *P* <0.001 by Wilcoxon). Felix™ isolated cells were also significantly more motile than those isolated by DGC (*P* <0.05), but not different from those recovered using the semen swim-up procedure.

**Figure 2:**
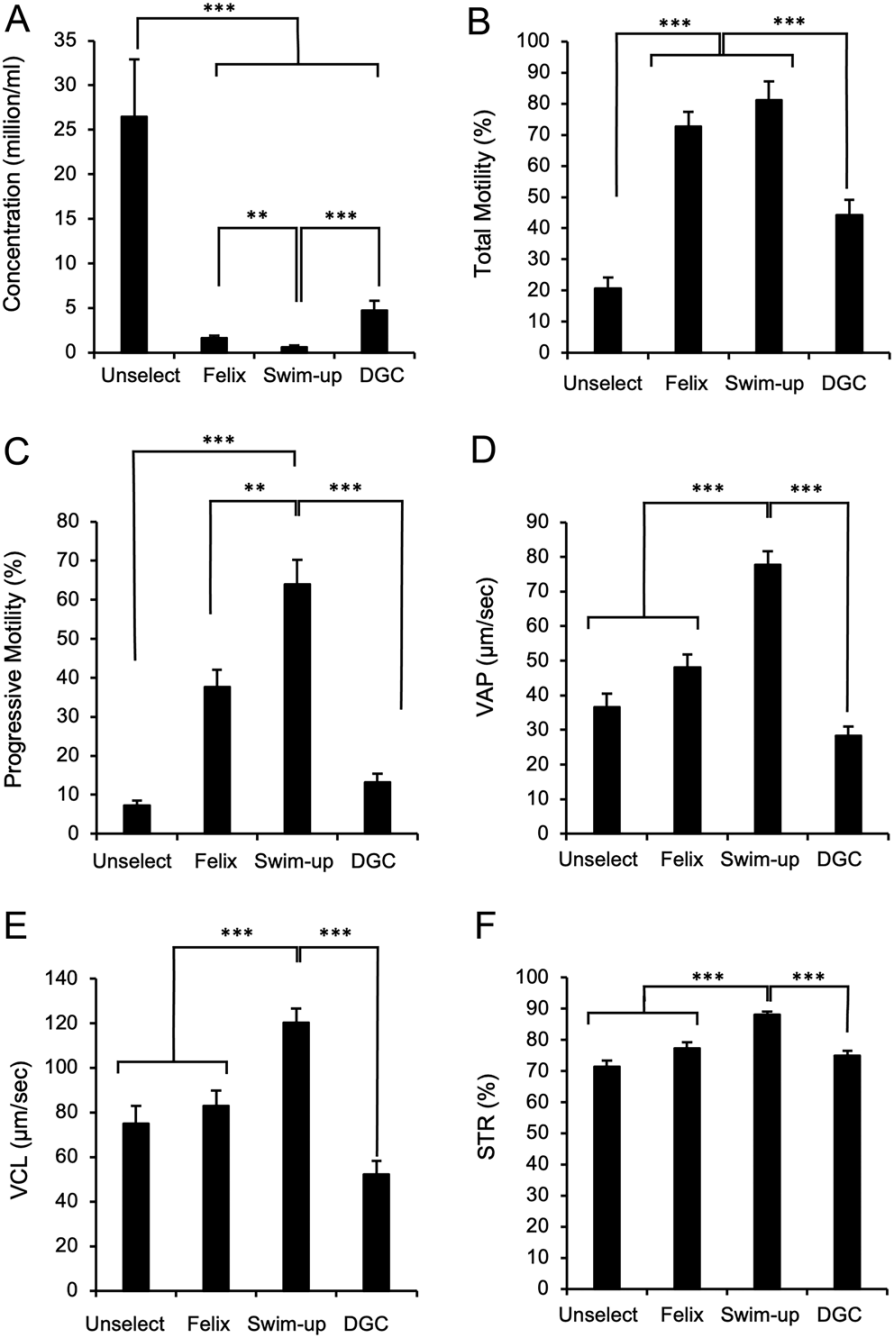
Impact of sperm isolation protocol on the movement characteristics of cryopreserved human spermatozoa. Following the freeze-thaw procedure, spermatozoa were recovered without any selection (Unselect) or following selection by the Felix™ electrophoretic system, swim-up, or DGC. (A) Concentration of recovered spermatozoa in millions/mL. (B) Percentage of motile cells. (C) Progressive motility. (D) VAP. (E) VCL. (F) STR. Full dataset is presented in Supplementary Table 2. Data presented as means ± s.e.; *n* = 15. **P < 0.01, ***P < 0.001.

In order to determine the impact of the sperm isolation procedure on the quality of sperm movement, an additional 15 specimens (Table 1) were analysed using a CASA system and the results are presented in Fig. 2. Within this group of samples, we saw the same pattern of sperm recovery as observed in the previous cohort of subjects, with swim-up generating a significantly lower number of cells than either Felix™ (*P* < 0.01) or DGC (*P* < 0.001). Similarly, total motility in the Felix™ and swim-up cohorts were significantly greater than the unselected and DGC-isolated populations but not significantly different from each other (Fig. 2B). However, when the quality of sperm movement was examined, it became clear that the swim-up methodology had selected a subset of highly motile cells that were significantly faster and more linear in their movement than those selected by DGC or Felix™. Thus, progressive motility (Fig. 2C; *P* < 0.001), VAP (Fig. 2D; *P* < 0.001), VCL (Fig. 2E; *P* < 0.001), VSL (data not shown; *P* < 0.001), STR (Fig. 2F; *P* < 0.001), and LIN (data not shown; *P* < 0.001) were all superior in the swim-up population compared with any other group. Furthermore, this superiority was maintained over a 3-h incubation period at 37°C (Supplementary Table 2). In addition, the Felix™ system was superior to DGC with respect to progressive motility (*P* < 0.001), VAP (*P* < 0.001), VCL (*P* < 0.01), VSL (*P* < 0.001), and ALH (*P* < 0.05) and again this advantage was sustained over a 3 h incubation period at 37°C (Fig. 2; Supplementary Table 2).

With respect to other aspects of semen quality, the Felix™ system was the only method to significantly improve vitality levels compared with the unselected controls (*P* <0.01) and the only technique to improve sperm vitality compared with DGC isolation (*P* <0.001), which was associated with the poorest levels of vitality (Fig. 1C). Sperm morphology was not significantly influenced by any of the sperm isolation methods employed in this study (Fig. 2D).

The sperm isolation techniques employed to recover cryostored human spermatozoa only impacted the tendency of the sperm mitochondria to generate ROS in the case of DGC-isolated cells where the percentage of live MSR-positive cells in the entire cell population was slightly reduced (*P* <0.05; Fig. 1E). This outcome may have reflected a loss of sperm viability in the DGC-isolated population because if the results were expressed as a percentage of live cells in the population, then no significant changes were observed (*P* > 0.05). The levels of lipid aldehyde formation in the spermatozoa were high following cryopreservation, with 37.4 ± 4.5% of cells 4-HNE positive in the unselected population. Sperm selection resulted in a reduction in the levels of 4-HNE expression in the case of Felix™ isolated cells compared with those prepared by DGC (*P* < 0.01, Fig. 1F). However, there was no significant difference between Felix™ and semen swim-up in terms of 4-HNE positivity (Fig. 1F).

**Figure 3:**
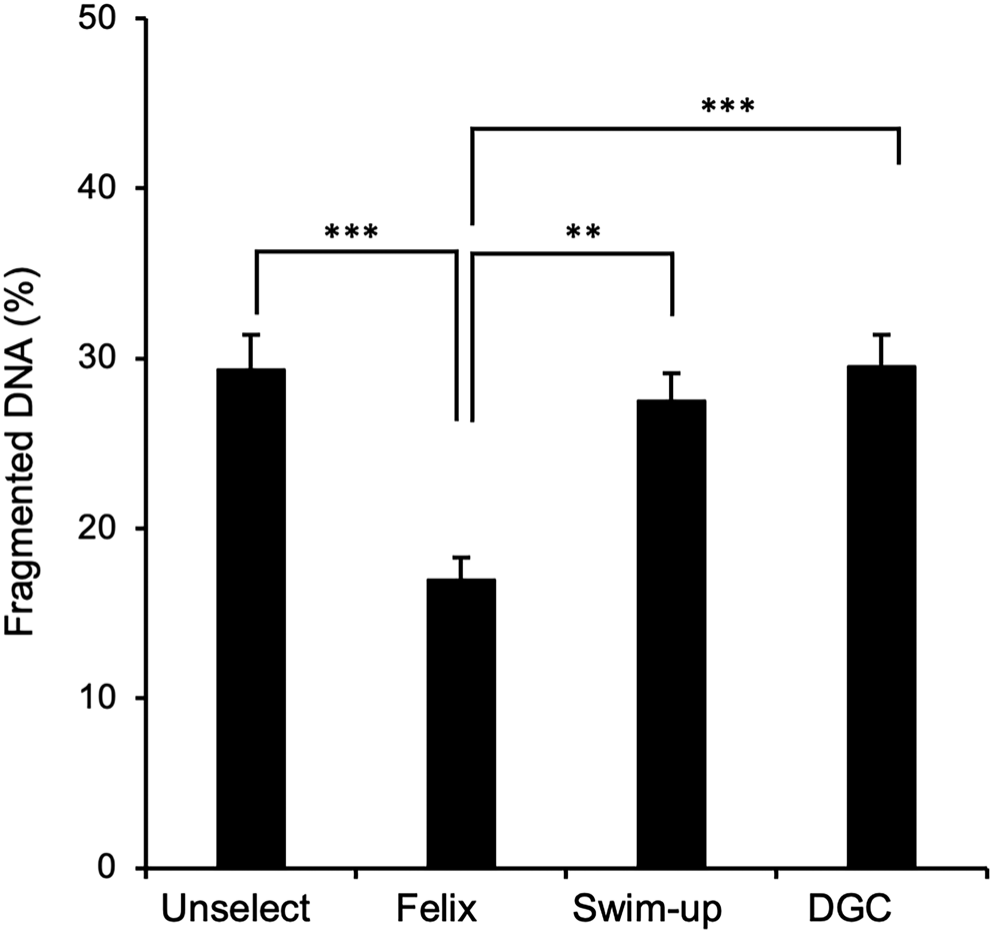
Impact of sperm isolation protocol on DNA integrity in cryopreserved human spermatozoa. Following the freeze-thaw procedure, spermatozoa were recovered without any selection (Unselect) or following selection by the Felix™ electrophoretic system, swim-up, or DGC. Data presented as means ± s.e.; *n* = 40. **P < 0.01, ***P < 0.001.

Isolation with the Felix™ device also had a significant impact on the levels of DNA damage observed after sperm isolation. In the unfractionated control as well as the spermatozoa isolated by semen swim-up or DGC, around 30% of the cells exhibited evidence of DNA damage. However, with the Felix™ system, the levels of DNA damage were approximately halved and significantly lower than those observed in the unselected population (*P* <0.001) or isolated by semen swim-up (*P* <0.01) or DGC (*P* <0.001) (Fig. 3).

## Discussion

The fundamental purpose of this study was to determine the optimum method for recovering human spermatozoa following cryostorage, for subsequent use in assisted conception therapy. Three different techniques were compared for this purpose: *semenswim-up*, the success of which depends on the intrinsic motility of the spermatozoa; *DGC,* which uses centrifugation through density gradient media to isolate the spermatozoa with the highest isopycnic density, and *the Felix™ system,* which isolates spermatozoa according to their size, motility, and net negative charge. It was hypothesised that the cryostorage process would present a particular challenge to these sperm isolation procedures given the extensive intracellular damage that is known to be induced by cryopreservation (Royere *et al.* 1996, Hammadeh *et al.* 2001*a*,*b*, Thomson *et al.* 2009, Le *et al.* 2019) as reflected in the observed loss of total motility, vitality, and DNA integrity.

Given the clear decrease in sperm quality precipitated by cryopreservation, isolating the best spermatozoa from the sample is a practical requirement for procedures that necessitate utilising healthy spermatozoa, such as IVF or intra-cytoplasmic sperm injection (ICSI) (Host *et al.* 2000). Damaged spermatozoa may influence the clinical outcome of these procedures by causing early embryonic death and miscarriage or, if the fetus goes to term, an increased risk of malformations, genetic mutations, and DNA imprinting-related syndromes (Bungum *et al.* 2007, Ricci *et al.* 2009, Gonzalez-Marin *et al.* 2012, Quinn *et al.* 2018, Aitken & Gibb 2022).

The results of this analysis revealed that the Felix™ system is more effective than either DGC or semen swim-up in isolating cryostored spermatozoa exhibiting low levels of DNA damage for use in assisted conception therapy. The Felix™ device has the advantage over DGC that it does not involve the application of centrifugal forces which might be particularly damaging to friable cryopreserved human spermatozoa. Furthermore, unlike the swim-up procedure, the Felix™ system is not entirely dependent on sperm motility, which is reduced as a consequence of the freeze-thaw process. Such an impact on motility explains why the sperm recovery rates with the swim-up procedure were significantly lower than those achieved with the Felix™ device or DGC (Fig. 1A and 2A) The one advantage of selecting cells solely on the basis of their motility, however, was that quality of sperm movement was significantly greater with the swim-up procedure than either Felix™ or DGC and, moreover, this difference was sustained for at least 3 h at 37°C *in vitro* (Fig. 2; Supplementary Table 2). As a result, it is conceivable that the swim-up procedure is optimal for IVF, providing sufficient cells can be recovered for fertilization. Felix™-isolated cells also exhibited significantly better motility than those prepared by DGC and had the advantage over both swim-up and DGC in reducing levels of sperm DNA damage. On this basis, the Felix™ system may be optimal for ICSI.

In addition to the quality of the isolated cells, one of the most important advantages of the Felix™ system is its relative efficiency. Both DGC and semen swim-up procedures required extensive processing times in order to prepare a population of spermatozoa for assessment. In the case of the semen swim-up protocol, the cells require incubation at 37°C for 30 min to 1 h, depending on the quality of the original sample, to achieve an adequate recovery of spermatozoa. The DGC technique was conducted at room temperature but involved exposure of friable human spermatozoa to the physical shearing forces generated during a total centrifugation time of 30 min and a total processing time of around 45 min. By contrast, the Felix™ system is conducted entirely at room temperature, does not expose spermatozoa to the shearing forces associated with centrifugation, and takes a total of 6 min from start to finish. This electrophoretic method, therefore, places less stress on the cells than either semen swim-up or DGC, and it may be for this reason that the DNA integrity of the spermatozoa is significantly better with Felix™ than any of the other sperm isolation protocols assessed. This advantage may be particularly important with cryopreserved spermatozoa that may be more susceptible to external stresses than fresh spermatozoa.

The freeze-thawing procedures used in this study are those approved by Monash IVF and are typical of the procedures employed in current clinical practice. They may not be optimal, however. For example, some authors have found that thawing at 40°C generates better sperm motility than the room temperature protocol used in this study (Calamera *et al.* 2010). Others have found that vapour fast freezing (Arciero *et al.* 2022) or vitrification (Isachenko *et al.* 2005, 2012) can also preserve the spermatozoa better than conventional cryopreservation procedures. In the future, it will be of interest to assess these innovative methodologies in conjunction with the Felix™ isolation procedure. Such studies could even include a reversal of the current procedure, whereby the spermatozoa are first isolated by the Felix device and then subjected to a process such as vitrification (Isachenko *et al.* 2005, 2012).

In terms of the underlying mechanisms responsible for damaging the spermatozoa during the freeze-thaw process, cryopreservation is known to enhance the levels of DNA damage seen in spermatozoa via mechanisms that appear to involve nucleases activated by apoptosis as well as oxidative stress (Paoli *et al.* 2014, Le *et al.* 2019). The involvement of oxidative stress in the induction of DNA damage during cryopreservation is also suggested by a plethora of studies indicating that antioxidants can reduce the levels of fragmentation observed (Paoli *et al.* 2014). It is traditional to view oxidative stress as originating from the exposure of human spermatozoa to excessive levels of ROS generated largely by the sperm mitochondria (Aitken *et al.* 1998, Koppers *et al.* 2008, Aitken & Gibb 2022). Interestingly, in this study, high levels of DNA damage were observed following cryopreservation that were not associated with an increase in mitochondrial ROS generation, although levels of 4-HNE generation were raised. Similarly, the low levels of DNA damage observed following recovery of the spermatozoa with the Felix™ device were not associated with a reduction of mitochondrial ROS generation although 4-HNE generation was reduced, particularly relative to DGC (Fig. 1E and F). For their part, the DGC-prepared populations clearly showed evidence of cellular damage relative to the Felix™-isolated cells as reflected in the paucity of their motility (Fig. 1B and 2) and vitality (Fig. 1C) and the relative abundance of 4-HNE positive cells (Fig. 1F) as well as DNA damaged cells (Fig. 3). At the same time, mitochondrial ROS generation was reduced (Fig. 1E). Thus, while there may an oxidative element to DNA damage following cryopreservation it does not, in the short term at least, involve increased ROS generation. An alternative explanation for these data is that cryopreservation is associated with the breakdown of existing lipid peroxides to aldehydes such as 4-HNE, possibly because of increased availability of transition metals such as iron and copper (Aitken *et al.* 1993). The concept that increased iron availability might be involved in the induction of sperm cryoinjury is suggested by the ability of lactoferrin to improve both stallion and ram sperm function following cryopreservation (Martins *et al.* 2018, Su *et al.* 2022). The addition of exogenous gangliosides has also been shown to protect frozen-thawed human spermatozoa from DNA damage partly via the ability of their sialic acid moieties to chelate transition metals such as iron and copper (Saladini *et al.* 2002, Gavella & Lipovac 2013). Moreover, many studies have shown that the addition of iron to sperm suspensions generates both lipid peroxidation and DNA damage via mechanisms that can be reduced with iron chelators such as deferoxamine and EDTA (Guthrie & Welch 2007). Since the negative charge carried by human spermatozoa, which enables them to become isolated in the Felix™ system, is known to be dependent on sialic acid residues (Ainsworth & Aitken 2011), it is possible that these negatively charged cells are protected from iron-mediated oxidative stress by virtue of the metal-chelating properties of their sialylated proteins and lipids (Saladini *et al.* 2002, Gavella *et al.* 2005). There is no doubt that human semen contains sufficient iron to promote the activation of destructive lipid peroxidation pathways and that chelation of such metals would have a beneficial impact on sperm function and DNA integrity (Kwenang *et al.* 1987, Huang *et al.* 2000, Hashemi *et al.* 2018). Moreover, in cryostored bovine semen, a negative correlation exists between seminal plasma iron content and DNA fragmentation (Zakošek Pipan *et al.* 2021). Further studies are now warranted to determine whether the negatively charged spermatozoa selected by the Felix™ system are protected from cryoinjury because their high sialic acid content limits the toxicity of transition metals released during the cryopreservation process (Schmid *et al.* 2013, Aitken *et al.* 2014, Gholirad *et al.* 2017, Chen *et al.* 2018).

## Conclusion

This is the first study to compare electrophoretic, DGC, and swim-up sperm isolation techniques on cryopreserved human spermatozoa. Each technique presents its own spectrum of advantages and disadvantages. With DGC, sperm recovery rates are high, but the spermatozoa are damaged in terms of their motility and DNA integrity. Swim-up, on the other hand, generates highly motile sperm populations, but recovery rates are extremely low and levels of DNA damage are not different from the unselected controls. By contrast, Felix™ significantly lowers the levels of DNA fragmentation observed in cryostored human sperm populations, while maintaining relatively high levels of motility and sperm recovery. Based on this evidence, Felix™ appears to be the optimal choice for recovering high-quality spermatozoa following cryopreservation for assisted conception purposes. This may be particularly useful, given the extensive use of cryostorage techniques for the long-term preservation of human fertility (Brezina *et al.* 2015).

## Supplementary Material

Supplementary Table 1. Attributes of semen quality and efficacy of sperm isolation according to location

Supplementary Table 2. Attributes of sperm movement in populations of cryopreserved human spermatozoa that have been frozen-thawed and isolated using swim-up, DGC or the Felix™ electrophoretic system and then incubated in vitro for 3 h at 37°C

## Declaration of Interest

RJA is Scientific Director of Memphasys Ltd and AH is a PhD student funded by Memphasys Ltd.

## Funding

Financial supports were provided by Memphasys Ltd for required equipment and reagents. A PhD scholarship was provided to AH.

## Author contribution statement

AH designed and conducted the entirety of the experiment and wrote and edited the resulting paper. Mentorship and manuscript editing was provided by RJA and HWB.
