# Supplementary material for: Analysis of sperm separation protocols for isolating cryopreserved human spermatozoa
Source: Reprod Fertil. 2023 May 2;4(2):e220133. doi: 10.1530/RAF-22-0133 (PMC10160538; doi:10.1530/RAF-22-0133)
Supplement: Supplementary Table 1. Attributes of semen quality and efficacy of sperm isolation according to location [file supplementary_table_1.pdf]

Supplementary Table 1. Attributes of semen quality and efficacy of sperm isolation according to location

Site	Treatment	Count (10^6/ml)	Motility (%)	Morph (%)	Vitality (%)	MSR (%)	4-HNE (%)	DNA frag (%)
Newcastle	Pre-freeze	58.2±8.7	47.0±3.9	7.4±0.6	77.8±2.5	-	-	-
	Post-freeze	15.9±4.1	17.6±3.4	6.7±0.7	39.0±3.3	9.8±1.07	40.3±5.7	28.3±2.6
	Felix	1.32±0.2	29.6±4.9	6.8±0.9	49.1±5.4	13.12±3.5	33.6±3.6	17.6±1.9
	Swim-up	0.5±0.2	31.6±6.0	6.6±0.8	40.3±6.2	12.2±2.0	36.1±3.7	30.3±1.8
	DCG	2.1±0.3	16.3±3.6	5.7±0.8	32.3±4.3	7.2±1.1	42.4±4.9	30.4±2.4
Sydney	Pre-freeze	76.9±15.3	66.9±2.7	6.0±0.8	84.9±4.1	-	-	-
	Post-freeze	20.5±4.5	16.4±3.5	5.8±0.8	34.0±3.4	17.4±4.9	32.2±7.3	31.1±3.6
	Felix	1.4±0.3	42.7±5.7	7.3±0.8	57.3±4.7	11.9±2.2	25.4±5.2	15.8±1.8
	Swim-up	0.5±0.1	30.1±6.4	5.5±1.0	40.8±6.4	11.4±2.3	33.7±5.4	23.0±3.1
	DCG	3.7±1.1	29.0±6.6	5.3±0.9	30.0±4.7	9.2±2.2	47.2±6.4	27.9±3.3

Least squares analyses

Sperm count: No significant impact due to location ($P > 0.05$), however a highly significant impact due to sperm isolation procedure ($P < 0.001$).

Sperm motility : Significant impact due to location for the prefreeze sample only ($P < 0.01$), however a highly significant impact due to sperm isolation procedure ($P < 0.001$).

Sperm morphology : No significant impact due to location ($P > 0.5$) or sperm isolation procedure ($P > 0.5$)

Sperm vitality : No significant impact due to location ($P > 0.05$), however highly significant impact due to sperm isolation procedure ($P < 0.001$) with Felix isolated cells exhibiting significantly better vitality than DGC ($P < 0.001$) but not spermatozoa isolated by swim-up.

Sperm mitochondrial ROS generation : No significant impact due to location ($P > 0.5$) and only a minor overall effect due to isolation procedure ($P < 0.5$).

Lipid peroxidation : No significant impact due to location ($P > 0.05$), but modest impact due to sperm isolation procedure ($P < 0.05$) with Felix exhibiting significantly less 4-HNE formation than samples prepared by DGC ($P < 0.05$).

DNA damage : No significant impact due to location ($P > 0.05$), however highly significant impact due to sperm isolation procedure ($P < 0.001$) with Felix significantly better than all other procedures ($P < 0.001$)
