# Supplementary material for: Analysis of sperm separation protocols for isolating cryopreserved human spermatozoa
Source: Reprod Fertil. 2023 May 2;4(2):e220133. doi: 10.1530/RAF-22-0133 (PMC10160538; doi:10.1530/RAF-22-0133)
Supplement: Supplementary Table 2. Attributes of sperm movement in populations of cryopreserved human spermatozoa that have been frozen-thawed and isolated using swim-up, DGC or the Felix™ electrophoretic system and then incubated in vitro for 3 h at 37°C [file supplementary_table_2.pdf]

Supplementary Table 2. Attributes of sperm movement in populations of cryopreserved human spermatozoa that have been frozen-thawed and isolated using swim-up, DGC or the Felix™ electrophoretic system and then incubated in vitro for 3 h at 37°C

Treatment	Count (10^6/ml)	Motility (%)	Progressive (%)	ALH (µm)	STR	VAP (µm/sec)	VCL (µm/sec)	VSL (µm/sec)
Post-freeze	26.5±6.5	20.6±3.7	7.2±1.4	3.8±0.4	71.4±2.0	36.7±3.9	75.1±8.0	28.6±3.6
Post freeze +3h	-	10.8±2.0†	3.1±1.0†	4.0±0.4	58.4±2.7†	31.0±2.9	71.0±5.4	20.9±2.7
Felix	1.6±0.3***	72.6±4.7***	37.6±4.5***	4.3±0.4	77.3±1.9*	48.0±3.8	82.8±7.1	41.2±3.4
Felix + 3h	-	65.8±6.1***	33.9±4.9***	4.6±0.2	76.1±2.7***	46.2±2.7*	80.9±4.0	38.4±2.9**
Swim-up	0.6±0.2***	81.2±6.2***	63.9±6.3***	5.3±0.4	88.0±1.0***	77.7±4.0***	120.3±6.4***	70.8±3.9***
Swimup+3h	-	66.3±7.5***	45.5±7.6***	6.2±0.3***	78.5±2.2†***	68.8±5.6***	122.6±8.4***	59.1±5.5***
DCG	4.7±1.1***	44.3±4.9**	13.2±2.2	3.0±0.3	75.0±1.6	28.4±2.7	52.4±5.9	23.0±2.1
DGC + 3h	-	28.8±3.8†**	12.0±2.3**	3.9±0.4	69.7±3.1**	38.0±3.7	70.3±6.9	28.9±3.8

*Indicates difference between the post-freeze control and sperm isolation procedure at the same time point. These data reveal the enhanced motility observed following sperm isolation with the Felix™ system or swim-up, compared with DGC. The ability of the recovered cells to sustain their motility following isolation with Felix™ or swim-up is similarly emphasized . *$P < 0.05$, **$P < 0.01$, ***$P < 0.001$.

† Indicates the ability of spermatozoa to sustain their motility compared with the same population of cells at T=0. These data indicate a significant loss of motility, progressive motility and STR over the 3 h incubation period if spermatozoa remained in the cryostorage medium and had not been subjected to any isolation procedure. Similarly, spermatozoa isolated by DGC lost motility over the 3 h incubation period. By contrast, spermatozoa isolated by swim-up or the Felix™ system maintained their motility advantage throughout their post-isolation incubation. †$P < 0.05$.
